# Impact of floral characters, pollen limitation, and pollinator visitation on pollination success in different populations of *Caragana korshinskii* Kom

**DOI:** 10.1038/s41598-019-46271-z

**Published:** 2019-07-05

**Authors:** Min Chen, Xue-yong Zhao

**Affiliations:** Northwest Institute of Eco-Environment and Resources, CAS, Lanzhou, China

**Keywords:** Ecology, Pollination

## Abstract

*Caragana korshinskii* Kom. has a significant function in desert-grassland revegetation in arid regions. Plant reproduction in arid regions can be restricted due to inadequate pollen receipt and reduced pollen transfer. An assessment of pollination success as a result of pollen limitation and pollinator visitation in various *C. korshinskii* populations is presently lacking. We thus tested three different treatments (pollen addition, control, and procedural control) to elucidate how pollen limitation affects seed numbers per flower in *C. korshinskii*. We also determined the effect of pollinator visit frequency on seeds per flower. Our results demonstrated that there was a higher proportion of open flowers and mature fruits in the managed population than in the natural population. Pollen addition significantly increased seed number per flower, and pollen limitation was determined to be a significant limiting factor in seed production. Furthermore, *Apis mellifera* was determined to be the principal pollinator, and pollinator visitation frequency was significantly correlated with open flower number. Our findings also demonstrated that pollinator visitation rate and seed production were positively correlated. Management and pollinator visitation could affect seed production, which may explain the higher seeds per flower in the managed population compared with the natural population.

## Introduction

As plants are immobile, they therefore depend on abiotic or biotic vectors to facilitate pollen transfer for sexual reproduction^1^, which has shaped floral attraction and plant mating systems^1^. Many studies have indicated that pollinator visits and behavior could affect the pollination success of plants^2,3^. Pollinator limitation can occur because flower visitors are either erratic or exhibit a preference for more attractive flowers^1,2^.

The pollination success of a plant can be hampered by an inadequate or insufficient supply of pollen, called “pollen limitation”^4^. There are both ecological and evolutionary determinants and consequences for pollen limitation^1^. In addition, many studies have measured the scale of pollen limitation based on the pollen limitation index (PL index) for each reproductive component^4,5^. There has been particular focus on pollen limitation because low pollen transfer and resource availability can impact seed production^1^. Pollen limitation is a widely-observed phenomenon that is typically interpreted as an indication of insufficient pollinator visitation in arid areas^6,7^. There is abundant evidence of pollen limitation due to insufficient pollinator services, particularly in animal-pollinated plants^8,9^.

Changes in habitat that cause increases or decreases in plant density may consequently alter pollinator availability and thus the pollination success of plants^10^. Human impacts on landscapes as well as grazing can also negatively influence pollinator visitor frequency^6^. Numerous plant species that rely on less effective pollinators may be subject to significant decreases in pollination success if pollinator activity is influenced by severe environmental conditions or climate change^11^.

*Caragana korshinskii* Kom. (Leguminosae: Ammopiptanthus) has an important function in the establishment of arid vegetation^12^. The aims of the present study were to (1) assess the floral trait differences between natural and managed populations, (2) establish the potential impact of pollen limitation on seeds per flower in both natural and managed populations and evaluate the correlation between pollinator visitation frequency and open flower number, and (3) determine how the seed production of *C. korshinskii* is impacted by pollinator visitation.

## Results

### Grazing effects on vegetation

**Figure 1:**
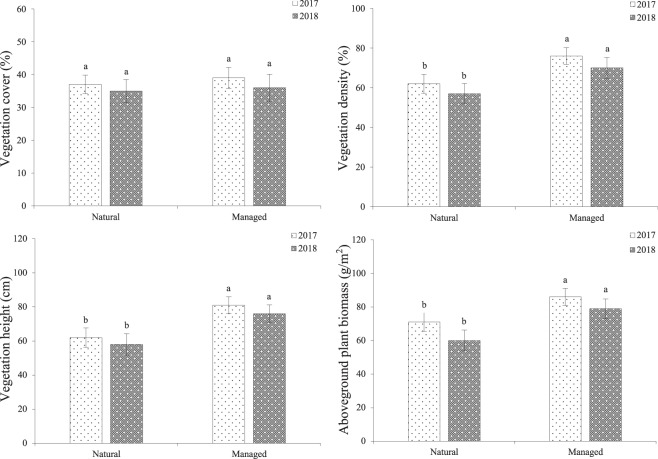
Grazing effects on vegetation between different populations. Vegetation cover (VC), vegetation density (VD), vegetation height (VH), and aboveground plant biomass (AGB) of *C. korshinskii*.

The vegetation cover (VC), vegetation density (VD), vegetation height (VH), and aboveground plant biomass (AGB) of *C. korshinskii* are indicated in Fig. 1. The findings show that the VD, VH, and AGB of the managed patch were significantly increased in comparison to those in the natural patch (*df* = 1, *P* < 0.05). However, no difference in VC was observed between the two studied patches (*df* = 1, *P* > 0.05).

### Floral traits

**Figure 2:**
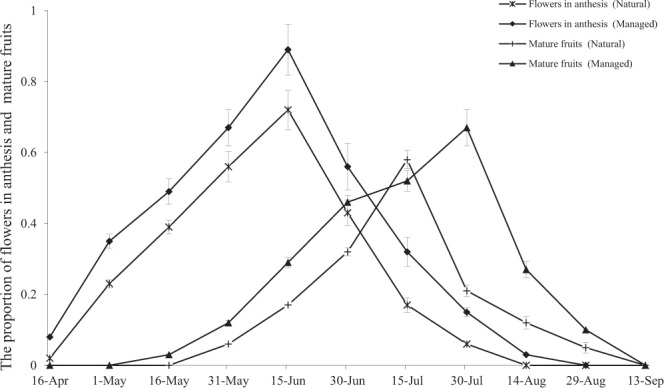
Phenology of *C. korshinskii* in natural and managed populations. The proportion of reproductive structures includes the mean number of flowers in anthesis and mature fruits. The mean number ± SE of phenological state structures per *C. korshinskii* individual in the reproductive season is illustrated.

Flowering generally took place from May until late June in both populations. At approximately 08:30, the flowers started to open and were completely open by 09:00. At approximately 14:00, the flowers started closing, and pollinator activity had ceased by 19:00 (Table 1). However, we observed that the managed population experienced a longer flower production period and flowering peak than the natural population (*P* < 0.05; Fig. 2).

**Table 1:** Flowering phenology of *C. korshinskii*.

Floral characters	Period
Flowering period	from May to June
Anthesis starts	08:00–08:30
Flowers completely open	09:00–11:00
Pollen release	08:30–14:00
Pollinator activity stopped	18:30–19:00

In the natural patch, the production peak of the mature fruits occurred in the third week of July. While generally similar patterns in mature fruit production were detected in the natural and managed populations, there were significant differences observed during the first week of August, with an increased number of mature fruits observed in the managed population (F = 18.36; *P* < 0.05; Fig. 2).

### Pollen limitation

**Figure 3:**
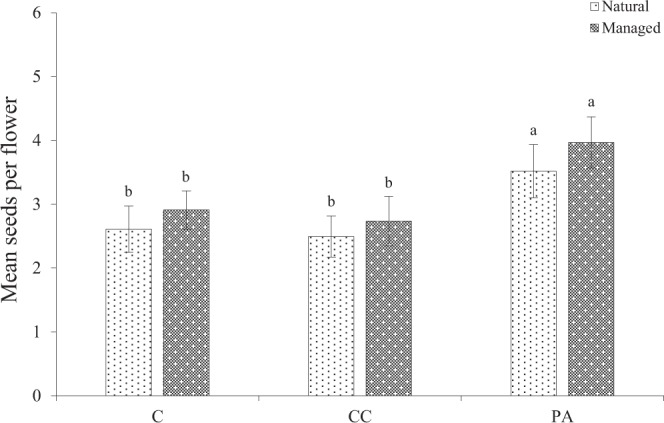
Mean seeds per flower under different treatments. Vertical bars indicate SE. Pollen-supplementation treatments: C, control; CC, procedural control; and PA, pollen added.

In the natural population, compared with the control and procedural control flowers, the mean number of seeds per flower was not significantly different in the natural population (Fig. 3), with the control values of 2.61 ± 0.8 (mean ± SE) and procedural control values of 2.49 ± 0.7 [Generalized linear Model (GLM), treatments effect, likelihood ratio χ^2^ = 0.89, *df* = 1, P > 0.05)]. In the managed patch, the mean seeds per flower, 2.90 ± 0.6 (control) and 3.97 ± 1.1 (pollen added), were significantly different between the control and pollen added treatment based on the GLM model (GLM, treatments effect, likelihood ratio χ^2^ = 31.24, *df* = 1, *P* < 0.001). Pollen supplementation was associated with a significant increase in mean seed production between the control and pollen added treatment in 2016–2018 in both patches (GLM, treatments effect, *df* = 1, *P* < 0.05; Table 2). Our findings demonstrated that the pollen limitation index in the natural population (0.29 ± 0.03) was more intense than in the managed population (0.27 ± 0.03).

**Table 2:** Impacts of the treatments (PA, C, and CC), different populations (natural and fragmented) and years (from 2016 to 2018) on seeds per flower of *C. korshinskii*. PA, pollen addition; C, control; CC, procedural control.

	Seeds per flower		
	likelihood ratio χ^2^	*df*	*P*
Years	0.057	1	0.81
Different populations	13.996	1	P < 0.001
Treatments	77.211	2	P < 0.001

### Pollinator visitation and activity

*Apis mellifera ligustica* Spin was the principal pollinator in the tested patched, pushing the petals of unopened flowers out to enter the flower. In addition, *A. mellifera* had successful visits because its hairy body could easily deposit more pollen per visit. Pollen release peaked between 10:00 and 14:00. *Apis mellifera ligustica* is active from 08:00 until 18:00, and the majority of the activity of *A. mellifera ligustica* overlapped with the open flowers of *C. korshinskii*. Of the 261 observed pollinator visits, *A. mellifera* accounted for 66.3% of the total visits. Other occasional visitors included *Bombus lucorum* L., *Serica orientalis* Motschulsky, *Anthophora fulvitarsis* Brulle, and *Eristalis cerealis* Fabricius, but these species only assisted in pollinator visits as they rarely touched the stigma and only visited infrequently.

**Figure 4:**
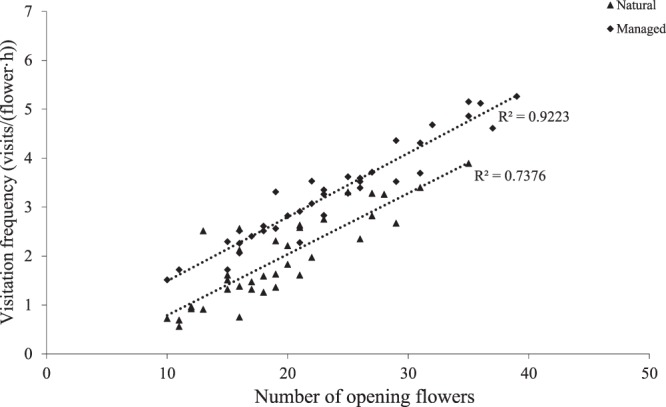
Relationship between visitation frequency and open flower numbers in the assessed patches.

The flowers of *C. korshinskii* have evolved a tripping mechanism. Pollinator activity is initiated with open flowers, with the pollinator acting as a tripping agent. We found that there was a significant correlation between the frequency of pollinator visits and the number of flowers that were open in both populations (natural, r = 0.96; managed, r = 0.86, *P* < 0.05; Fig. 4). Furthermore, our findings demonstrated that the V_f_ of *A. mellifera ligustica* in the managed population (5.26 ± 0.6 visits/hour) was significantly higher than V_f_ in the natural population (3.89 ± 0.5 visits/hour; *df* = 1*, P* < 0.05).

### Pollinator visitation effects on seed production

**Figure 5:**
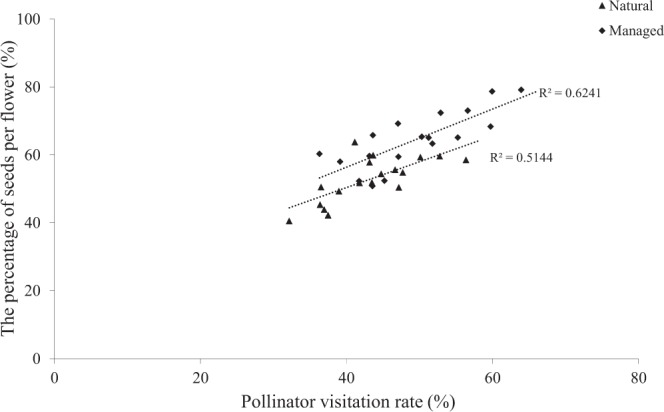
Relationship between the amount of seeds per flower and the pollinator visitation rate for *C. korshinskii* in the assessed patches.

Of the flowers in the natural patch, effective pollinators visited (V) 43.62% at least once, and 25.13% generated seeds (S), which resulted in the visited flowers (S/V × 100%) having a seed production percentage of 57.61%. Our findings demonstrated that in the managed patch, 49.36% of the flowers had been visited and 30.80% produced seeds, which resulted in the visited flowers having a seed production of 62.39%. These findings illustrated that there was a significant correlation between the seed production percentages among the visited flowers and the pollinator visitation rates both in the natural patch (the percentage of seeds: r = 0.72, *P* < 0.01) and the managed patch (the percentage of seeds: r = 0.79, *P* < 0.01; Fig. 5).

## Discussion

### Floral traits influencing pollinator visitation and activity

Floral traits impact pollinator activity and visitation and also affect pollination efficiency^13,14^. Generalist pollinators are subject to a high degree of environmental variation, and thus pollinator learning can potentially have a significant function in plant-insect coevolution^12^. Ortíz *et al*. (2010) demonstrated that flower resource density could be associated with the variation in visiting frequency and pollinator behavior^15^. In particular, nectar and pollen are the targets of pollinators^16,17^. We discovered that the proportion of open flowers could be significantly impacted by management, with the flowers in the managed population possessing more floral resources than those in the natural population. These findings might provide an explanation as to why a positive relationship exists between pollinator visiting frequency and the number of flowers that are open.

The likelihood of successful pollen vectors visiting a plant increases with pollinator activity^18^. The convergent evolution of floral traits with the traits of their common pollinators is widespread in plants and constitutes one of the most visual demonstrations of natural selection^19^. In arid regions, a high frequency of pollinator visits is more efficient because the filaments of flowers dry easily. In addition, pollinator visits are sometimes erratic and less persistent partly as a result of strong winds and high temperatures, which could severely impact pollinator activity^11^. In the present analysis, complete opening of the flowers was observed between 08:00 and 14:00, which enabled pollen release and represented a significant period for successful pollinating of *C. korshinskii*. Furthermore, the greatest *A. mellifera* activity overlapped with this period. Therefore, *A. mellifera* had the highest visiting frequency in comparison to the other pollinators.

### Pollen limitation and pollinator visitation in different populations

Plant life-history and the mating system may be correlated with the likelihood or degree of pollen limitation^20^. In many flowering plants, pollen limitation affects processes from pollination to seed production^1,19^ (Ashman *et al*. 2004; Ryan & David 2013). Pollen limitation is believed to have occurred if pollen supplementation increases fruit or seed production^20,21^. We discovered that pollen addition significantly increased the seeds per flower in the present study, indicating that seed production was pollen-limited in this species.

Earlier reports suggested that pollen quantity limitation is associated with both the pollinator frequency and pollinating effectiveness^1^. The foraging patterns of pollinators can be altered by management and human impacts on habitats, affecting pollinator behavior^16,22^. In the majority of angiosperms, pollinator activity is declining as a result of reduced floral rewards (nectar or pollen) and a lack of nesting requirements for pollinator species^23,24^. The results of the current study showed that grazing significantly influences vegetation density, vegetation height, and aboveground plant biomass in natural patches. In addition, management could significantly affect the peak flowering period and the proportion of open flowers.

Pollen limitation is significantly associated with pollinator activity and visitation, and pollen limitation typically takes place when incompatible pollen is deposited when pollinators are infrequent due to decreased floral resources^5,9,25^. Elucidating the association between pollen limitation and pollinator visitation across populations could be necessary for improving our ability to predict decreased pollinators and seed production in conserved plant populations^26,27^. In addition, a positive association between pollinator visitation frequency and open flower numbers was detected in both populations. Additionally, the flower density was greater in the managed than in the natural population. Therefore, the managed plants could attract more pollinator visits than the natural plants as a result of increased floral resources in the former.

### Pollinator visitation affects the pollination success of *C. korshinskii*

The sexual reproduction of seed plants is dependent on pollination, and pollination by insects appears to be dominant in angiosperms^28^. Reproductive success or failure is greatly dependent on pollinator activity and frequency^29^. Pollination has recently been shown to be crucial for the sexual reproduction of seed plants, with pollinator visiting frequency constituting an important predictor of pollination success^30,31^. The main biotic factors influencing pollination success include pollinator visitation, pollen quality, and reward systems^32^.

In arid regions, overgrazing is considered as a significant contributor to grassland degradation^33^. Furthermore, trampling by livestock erodes the crust and soil aggregate stability, while livestock feeding decreases vegetation cover^34^. Management and habitat changes also can affect pollinator visitation, which could further alter the outcrossing success of plants^35^. An earlier study indicated a reduction in pollinators is associated with a decreased quantity of pollen supplied to the stigmas and a reduction in cross-pollen transfer probability, thus causing decreased seed production^2^. Arid environments are associated with an increased frequency of unreliable and persistently less abundant pollinators partly as a result of grazing and habitat fragmentation, which severely hamper pollinator visits^16^. Decreased numbers of pollinators limit cross-pollen transfer probability, which might disturb the pollination process of plants, thus altering the foraging patterns of pollinators and contributing to the decline in plant populations^22,36^. When moderate grazing management was performed, we found that grazing had a significant impact on plant resources in the process of pollination.

In *C. korshinskii*, insect pollination plays a dominant role in the breeding system^37^. Moreover, *A. mellifera* tended to visit regions with more resources and stayed longer in the managed population. The findings of the present study also confirm that increased pollinator visitation rates are associated with increased seed production.

## Methods

### Species

*Caragana korshinskii* is a shrub that typically ranges between 0.4 and 2.0 m in height and occurs primarily in the provinces of western Inner Mongolia and Gansu. The root system of *C. korshinskii* is well-developed and drought resistant. This species is also valued for its medicinal properties^38^.

### Study area and experimental design

**Figure 6:**
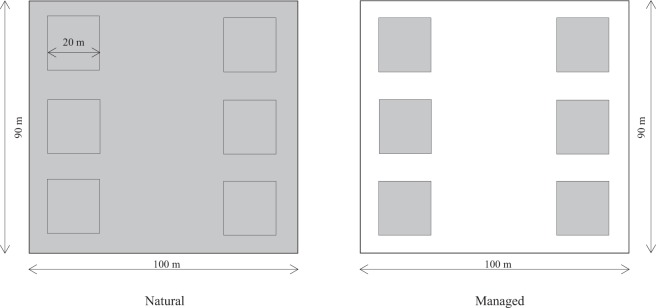
The experimental design comprised two patches and 12 plots, six natural plots, and six managed plots (20 × 20 m). In the natural patch, grazing experiments were performed. In addition, natural plots were arranged symmetrically and enclosed by undisturbed vegetation (gray area). Species other than *C. korshinskii* were removed from the managed patch (white area). Water was provided in the managed patch.

The study site was situated in the Urat Desert grassland (Fig. 6) to the north of Yinshan Mountain (41°06′–41°25′ N and 106°59′–107°05′ E). Based on the meteorological data obtained from the Urat Desert Grassland Research Station, the average annual temperature, average annual potential evapotranspiration, and average annual rainfall is 5.6 °C, about 2200 mm, and about 153.9 mm, respectively.

This experiment included two patches with six natural plots and six managed plots of 20 m × 20 m each. The studied plots were originally desert-grassland, with *C. Korshinskii* being the naturally dominant species in the six plots without artificial management. *Reaumuria songarica* (Pall.) Maxim was an associated species in the natural patches. In the natural patches, grazing experiments were performed from 2016 to 2018. Based on the grazing capacity of the desert steppe in Inner Mongolia, a moderate grazing intensity (0.25 sheep per ha) was established. Grazing ensued daily from May to August during the grazing period (from 07:00 to 19:00), and the sheep were placed in an enclosure during nighttime. In the managed patches, *C. korshinskii* was also the dominant species, and other species were repeatedly cleared (five times per year). In addition, we protected these plots from livestock grazing and also provided water. The natural and managed patches were separated by approximately 1 km to avoid the mutual interference of pollinators. In both patches, the average plant density was 15 individuals per 100 m^2^ (Fig. 1).

### Floral traits

In the studied patches, we randomly labeled 50 flowers to observe the anthers and buds. Floral phenology (anthesis and pollen dehiscence) was investigated with bagged flower buds and observed from 06:00 until senescence. In each population, the phenological characters of 10 individual *C. korshinskii* plants per plot were given labels and studied. On each branch, the flowers in anthesis and fruits on branches were enumerated during the entirety of the reproductive season (from May to August) for each plant. The proportion of flowers in anthesis and the numbers of mature fruits were then calculated.

### Pollen limitation

In order to calculate the degree of pollen limitation, a pollen supplementation experiment was set-up during flowering. Three treatments were established to assess the impact of pollen limitation on seeds per flower: control, procedural control, and pollen addition.

Eighteen healthy plants were labeled in each patch, and one inflorescence on each labeled plant was marked. Fresh pollen from plants situated 15 m distant or farther from the 18 experimental plants was obtained for pollen supplementation (six plants per plot) until sufficient pollen grains were obtained. For 12 labeled plants, we labeled eight flowers on each plant separate from the marked inflorescence and added outcrossed pollen to 4 flowers as the pollen-added treatment. Another 4 flowers in the same inflorescence were left untouched as controls. In the remaining six plants, four flowers from the center of the inflorescence of each plant were also marked and constituted the procedural control (CC treatment).

In August, we collected and counted all seeds per flower from control and pollen-added treatments. In this study, we used the seeds per flower to calculate pollen limitation. The following equation was used to calculate the pollen limitation index for the controls:

1 $${{\rm{PL}}}_{{\rm{C}}}={\rm{1}}\,-\,({{\rm{RS}}}_{{\rm{C}}}/{{\rm{RS}}}_{{\rm{PA}}})$$

Where RS_C_ and RS_PA_ are seeds per flower in the control and pollen-added treatment, respectively. Positive values of pollen limitation indicate a high intensity of pollen limitation, whereas negative values indicate the opposite^39^.

### Visitation frequency and pollinator activity

To conduct surveys of the pollinators in the 12 plots (six plots per patch), we selected 72 healthy plants in the studied patches. For each selected plant, 10 flowers were labeled with tags at the bud stage. These marked flowers were observed daily between 07:00 and 19:00. Pollinators collecting nectar and pollen were recorded, and pollinator visit duration was measured using digital audio tape recorders. The quantity and identity of floral visitors from May to August were determined, and pollinators were captured with insect nets. In addition, we determined that effective pollination had occurred when pollinators collected pollen or delivered it to the stigmas^37^. Pollinators that visited the flowers were sampled for identification in the laboratory. We recorded the visiting frequency of the pollinators (V_f_), which was calculated based on the following equation^40^:

2 $${\rm{V}}{i}{\rm{siting}}\,{\rm{frequency}}=\frac{{\rm{Number}}\,{\rm{of}}\,\mathrm{visits}\,}{{\rm{Number}}\,{\rm{of}}\,{\rm{flowers}}\ast {\rm{Observation}}\,\mathrm{time}\,}$$

### Pollinator visitation affect seed production

To assess the impact of pollinator visitation on seed production, 18 plants in the natural patch and 18 plants in the managed patch were labeled during flowering. Ten flowers from each plant were randomly selected and tagged. The flowering stage and growth development of the labeled flowers were determined from the film recordings. Furthermore, the proportions of flowers that were open and the pollinator visits in the period May–August were recorded. We observed that the effective pollinators carried large quantities of pollen grains during their visits. In addition, these species had a higher visiting frequency than the other pollinators. The number of visited flowers and seed production were recorded when all seeds were mature. Additionally, the visited flower and mature seed percentages were calculated in accordance with the following equation^24^:

3 $${\rm{Percentage}}\,{\rm{of}}\,{\rm{seeds}}\,{\rm{among}}\,{\rm{visited}}\,{\rm{flowers}}=\frac{{\rm{S}}}{\,V\,}\times {\rm{100}} \% $$

Where S and V represent the proportion of flowers producing seeds and the proportion of visited flowers, respectively.

### Data analyses

A GLM was used to assess the impacts of pollination treatments (C, CC and PA), population types and years (from 2016 to 2018) on seeds per flower^41^. A gamma distribution and a logit link function were implemented in the model. The pollination treatments, population types, and years constituted the fixed factors, and the mean seeds per flower represented the dependent variable in the model. The likelihood ratio test was applied in the model, and treatment differences were assessed with Tukey’s multiple comparisons.

Analysis of variance (ANOVA) was used to evaluate the production of open flowers and mature fruits in both populations. Regression analyses were performed in SPSS 21.0^42,43^. In addition, regression analyses using the number of open flowers as the independent variable were also performed, with pollinator visitation frequency as the dependent variable in this model.
